# Integrative analysis of DNA methylation and gene expression identified cervical cancer-specific diagnostic biomarkers

**DOI:** 10.1038/s41392-019-0081-6

**Published:** 2019-12-13

**Authors:** Wanxue Xu, Mengyao Xu, Longlong Wang, Wei Zhou, Rong Xiang, Yi Shi, Yunshan Zhang, Yongjun Piao

**Affiliations:** 1 School of Medicine, Nankai University, Tianjin, China; 2 Tianjin Key Laboratory of Human Development and Reproductive Regulation, Nankai University Affiliated Hospital of Obstetrics and Gynecology, Tianjin, China; 3 Reproductive Medical Center, Nankai University Affiliated Hospital of Obstetrics and Gynecology, Tianjin, China

**Keywords:** Genome informatics, Tumour biomarkers

## Abstract

Cervical cancer is the leading cause of death among women with cancer worldwide. Here, we performed an integrative analysis of Illumina HumanMethylation450K and RNA-seq data from TCGA to identify cervical cancer-specific DNA methylation markers. We first identified differentially methylated and expressed genes and examined the correlation between DNA methylation and gene expression. The DNA methylation profiles of 12 types of cancers, including cervical cancer, were used to generate a candidate set, and machine-learning techniques were adopted to define the final cervical cancer-specific markers in the candidate set. Then, we assessed the protein levels of marker genes by immunohistochemistry by using tissue arrays containing 93 human cervical squamous cell carcinoma samples and cancer-adjacent normal tissues. Promoter methylation was negatively correlated with the local regulation of gene expression. In the distant regulation of gene expression, the methylation of hypermethylated genes was more likely to be negatively correlated with gene expression, while the methylation of hypomethylated genes was more likely to be positively correlated with gene expression. Moreover, we identified four cervical cancer-specific methylation markers, cg07211381 (RAB3C), cg12205729 (GABRA2), cg20708961 (ZNF257), and cg26490054 (SLC5A8), with 96.2% sensitivity and 95.2% specificity by using the tenfold cross-validation of TCGA data. The four markers could distinguish tumors from normal tissues with a 94.2, 100, 100, and 100% AUC in four independent validation sets from the GEO database. Overall, our study demonstrates the potential use of methylation markers in cervical cancer diagnosis and may boost the development of new epigenetic therapies.

## Introduction

Cervical cancer is one of the most frequently diagnosed cancers and the major leading cause of cancer death in women, with >500,000 cases and 300,000 deaths each year worldwide.^1^ Although the death rate from cervical cancer has decreased over the past few decades in several populations in North America, Europe, Australia, and New Zealand, the mortality of cervical cancer has increased rapidly in populations in Eastern Europe, Central Asia, and Africa due to the lack of effective screening and low rates of vaccination.^2^ Most cervical cancers are caused by human papillomavirus (HPV), but immunosuppression, smoking, pregnancy history, and long-term use of oral contraceptives have also been reported as risk factors for cervical cancer.^3^ New strategies, such as next-generation sequencing, are providing unbiased opportunities to uncover the genetic etiology of cervical carcinogenesis. Genetic^4–6^ and epigenetic^7–9^ variations may alter the expression of oncogenes or tumor-suppressor genes in cervical cancer.^10^ The 5-year survival rate of cervical cancer patients detected at an early stage is 92%.^2^ However, the survival rate decreases dramatically if the cancer cells spread to surrounding tissues or other parts of the body. Therefore, the early detection of cervical cancer is an urgent need for physicians to improve both treatment and outcomes and to enhance early intervention and consultation for patients to improve their quality of life.

The Pap smear, also called the Pap test, is an exam that has been widely used in the clinic for screening for the presence of precancerous or cancerous cells on the cervix. However, a Pap smear shows a low sensitivity and a high false-negative rate.^11^ The HPV test is also a recommended screening tool for cervical cancer that can not only detect the presence of HPV but also determine the subtype. Although a number of studies^12–15^ have shown that HPV screening is more sensitive than the Pap test in the detection of high-grade cervical carcinoma, HPV testing is not capable of distinguishing the true triggers and linked factors to provide an accurate prediction, as not all subtypes of HPV infection could lead to cervical cancer. Molecular markers have been identified to improve the capability for risk prediction, early detection, and prognosis prediction of cervical cancer. Squamous cell carcinoma antigen (SCC-Ag),^16^ cancer antigen 125 (CA-125),^17^ cancer antigen 19-9 (CA19-9),^18^ and cytokeratin 19 fragment antigen 21-1 (CYFRA 21-1)^19,20^ are clinically available tumor markers for the diagnosis and prognosis of cervical cancer. Although an increasing number of markers, such as keratin 4 (KRT4), keratin 17 (KRT17), CD28, PTEN, miR-29a, miR-21, and HPV E4, are being investigated continuously,^21–23^ the actual usage rate of these markers in clinical practice is very low. Thus, finding new tumor markers to improve both the sensitivity and specificity of cervical cancer diagnosis is of great importance.

DNA methylation is a major epigenetic mechanism that involves the transfer of a methyl group to the C5 carbon residues (5mC) of cytosines that is mediated by a family of DNA methyltransferases.^24^ DNA methylation plays important roles in various biological processes,^25^ including the regulation of gene expression,^26^ genomic imprinting,^27^ cell differentiation,^28^ development,^29^ and inflammation.^30^ Aberrant methylation has been reported to be associated with various diseases, including cancers.^31^ Most of the CpGs in CpG islands are generally unmethylated in normal cells. Hypermethylation of those CpGs is one of the commonly observed alterations in tumor cells, which may lead to the silencing of tumor-suppressor genes.^32^ For example, p14ARF promoters have been found to be hypermethylated in colon cancer.^33^ The BRCA1 and TMS1 promoters were observed to be hypermethylated in breast cancer cells,^34^ and DAPK and RASSF1A were hypermethylated in lung cancer.^35^ Several studies^36,37^ have reported that methylation markers are more sensitive than protein markers, and thus, cancer-specific methylation markers have great potential to be used to accurately diagnose cancers in clinics.

In this study, we integrated Illumina HumanMethylation450K methylation data and RNA-seq gene expression data from The Cancer Genome Atlas (TCGA) to identify cervical cancer-specific DNA methylation markers. By using a systemic screening method and a machine-learning approach, we identified four cervical cancer-specific methylation markers with a sensitivity of 96.2% and a specificity of 95.2% with the tenfold cross-validation of TCGA data. The four markers could distinguish tumor from normal tissues with 94.2, 100, 100, and 100% area under the curve (AUC) values in four independent validation sets from the Gene Expression Omnibus (GEO) database.

## Results

### Unsupervised clustering analysis of DNA methylation in cervical cancer

**Fig. 1:**
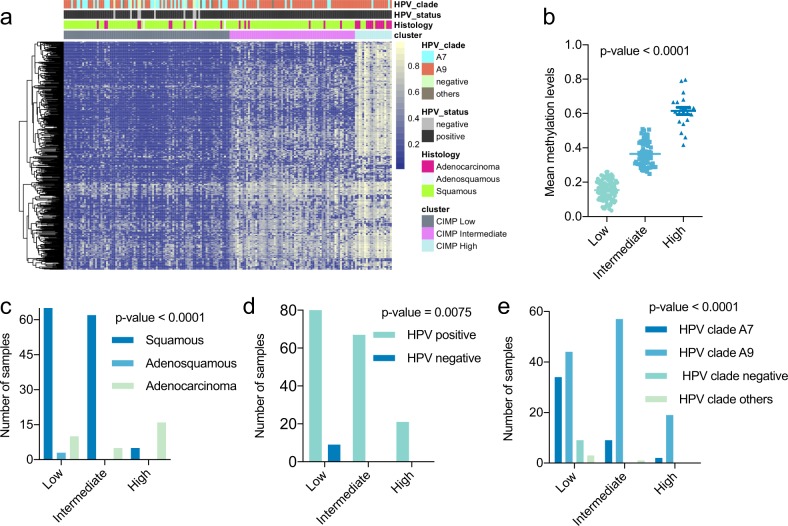
The DNA methylation landscape of cervical carcinoma. **a** Unsupervised clustering of methylation levels in cervical cancer. Samples are presented in columns, and the 591 most variable CpG loci (mean methylation level *β* < 0.05 in normal samples and a standard deviation *σ* > 0.20 in tumor samples) are presented in rows. The three identified clusters were denoted as CIMP-high (*n* = 20, CpG island methylator phenotype), CIMP-intermediate (*n* = 69), and CIMP-low (*n* = 89). Primary tumor features significantly associated across the three clusters (Fisher’s exact test *p*-value <0.001) are indicated at the top of the heat map. **b** Differences (*p*-value < 0.0001) in the methylation levels of the three consensus clusters. The CIMP-high group exhibited significant hypermethylation compared with the other groups. **c**–**e** The sample distributions in terms of HPV status, histology, and HPV clade in the three clusters are presented in **c**–**e**, respectively

Of 307 TCGA cervical tumor samples, 178 samples (Table S1) with well-reported clinical information were used for the clustering analysis. Consistent with previous studies,^1^—consensus clustering of 591 of the most variable DNA methylation probes identified three clusters that were designated as CIMP-high (CpG island methylator phenotype), CIMP-intermediate, and CIMP-low (Fig. 1a). Twenty (11%), 69 (39%), and 89 (50%) samples were clustered as CIMP-high, CIMP-intermediate, and CIMP-low, respectively. The average methylation levels among the most variable CpGs differed significantly (*p*-value <0.0001) between clusters, with the CIMP-high group showing the highest methylation level and the CIMP-low group showing the lowest level of methylation (Fig. 1b). Three clinical features, including histology (*p*-value = 3.794e–09), HPV status (*p*-value = 0.007229), and HPV species (*p*-value = 7.954e–06), were found to be associated across the three clusters (Fig. 1a). Among the tumor samples, there were 31 (17%) adenocarcinomas, 144 (81%) squamous cell carcinomas, and 3 (2%) adenosquamous carcinomas. In terms of HPV infection, there were 169 (95%) HPV-infected tumors, including 120 infected by A9 species, 45 infected by A7 species, and 9 (5%) tumors that were not infected by HPV. Most of the adenocarcinomas were enriched in the CIMP-high cluster, while the CIMP-low and CIMP-intermediate groups had more squamous cell carcinomas (Fig. 1c). Adenosquamous cancers were only found in the CIMP-low group. All patients in the CIMP-high and CIMP-intermediate clusters were infected by human papillomavirus (HPV), while patients without HPV infection were distributed within the CIMP-low group (Fig. 1d). HPV A7 types were enriched in the CIMP-low cluster, and most samples in the CIMP-high group were infected by HPV A9 (Fig. 1e).

### Differential methylation and expression analysis

**Fig. 2:**
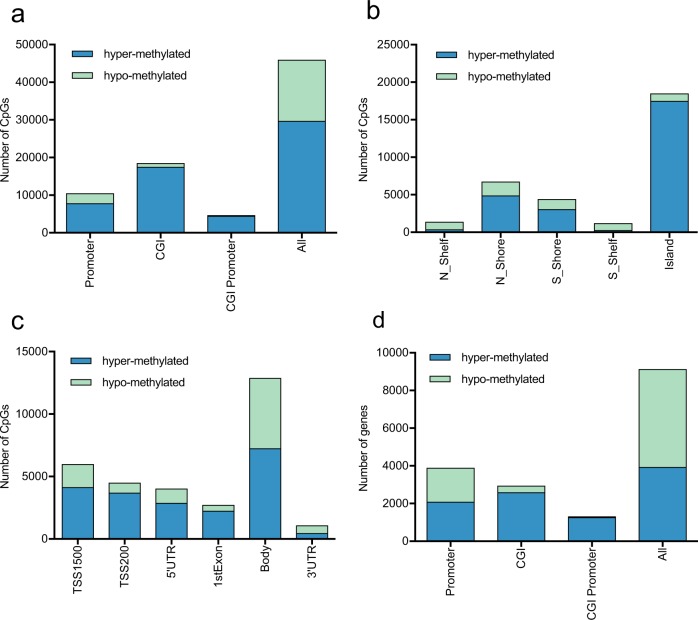
The number of hypo- and hypermethylated CpGs and genes. **a** Distribution of DMCs in different genomic locations, including promoters (1500 bp upstream of TSSs), CpG islands (CGI), promoters within CpG islands (CGI promoter), and the whole genomic region (all). **b** Distribution of DMCs in different regions related to CGIs, including CpG islands, CpG shores, and CpG shelves. **c** Distribution of DMCs across gene regions (TSS1500, TSS200, 5′ UTRs, first exons, gene bodies, and 3′ UTRs). **d** Distribution of DEGs in different genomic locations

A total of 46,040 CpGs were differentially methylated between the 113 normal and 307 tumor samples. There were 29,730 hypermethylated and 16,235 hypomethylated CpGs in cervical carcinoma. We then examined the distribution of the differentially methylated CpGs (DMCs) in various functional genomic regions, such as promoters, CpG islands (CGIs), and CGI promoters (Fig. 2a). In assessing the whole genome, 65% of CpGs were hypermethylated, and 35% of CpGs were hypomethylated. Increased hypermethylation was observed in promoters, CGIs, and CGI promoters (7851, 17,515, and 4539, respectively). By considering the CpG content and the neighboring context (Fig. 2b), the hypermethylation rate of CpG islands was shown to be the highest (95%), followed by that of N-Shore (72%), S-Shore (70%), N-Shelf (27%), and S-Shelf (23%). Examining the sites surrounding genes revealed that the hypomethylation rate was high in the 3′ UTR, while the hypermethylation rate was high in the regions near transcription start sites (TSS), such as TSS200, TSS1500, 5′ UTRs, first exons, and gene bodies. Then, we mapped the DMCs to genes, identifying 3939 hypermethylated genes and 5197 hypomethylated genes. The number of hypomethylated genes was higher than that of hypermethylated genes in the whole genome, while an increased number of hypermethylated genes was observed in the functional genomic area (Fig. 2d). Differential expression analysis was performed between the 69 normal and 304 tumor samples. A total of 4949 differentially expressed genes (DEGs) were detected, with 3096 upregulated genes and 1853 downregulated genes.

### Impact of DNA methylation on gene regulation in cervical cancer

**Fig. 3:**
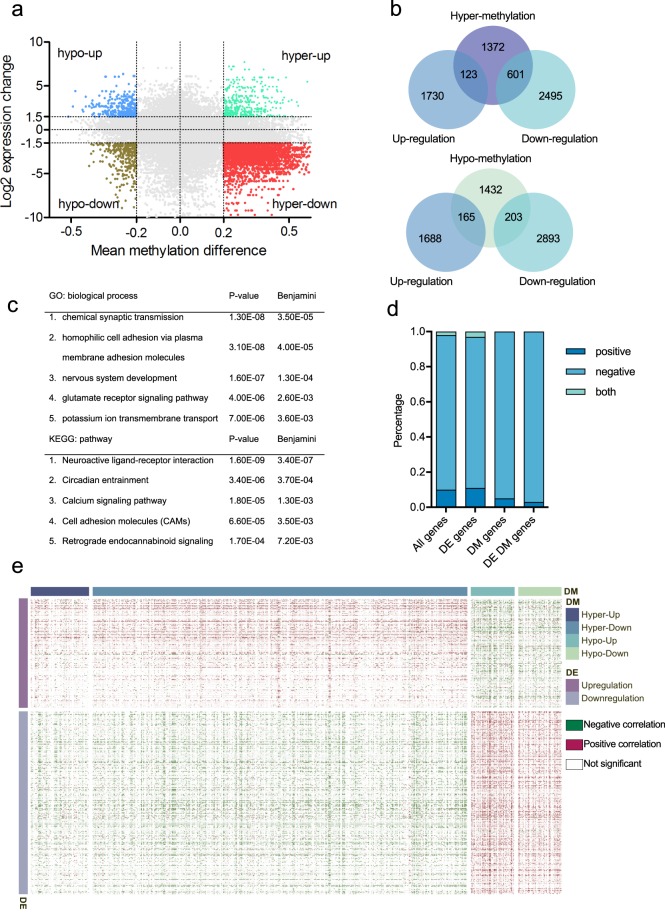
Integrative analysis of DNA methylation and gene expression. **a** Scatter plot of mean methylation difference versus log2 expression change. Each point represents a CpG-gene pair. **b** Venn diagrams summarizing the intersection between hypermethylated genes and DEGs (top) and between hypomethylated genes and DEGs (bottom). A gene was considered to be differentially methylated if there was at least one DMC in its promoter region. **c** Representative gene ontology (GO) terms and Kyoto Encyclopedia of Genes and Genomes (KEGG) pathways enriched in hyper–down genes. The functional annotation analysis was conducted by using DAVID, and the top five biological processes and pathways are reported with their p-values and Benjamini–Hochberg values. **d** Correlation between DNA methylation and gene expression (local regulation). Pearson’s correlation coefficient was calculated for all genes, DEGs, DMGs, and differentially expressed and methylated genes. The cutoffs for a significant correlation were $|{\mathrm{\gamma }}$| >0.3 and an adjusted *p*-value < 0.05. **e** Correlation between DNA methylation and gene expression (distant regulation). The Pearson correlation was calculated between 1092 CpGs in differentially expressed and methylated genes and 4949 DEGs

The integrative analysis of DNA methylation and gene expression was conducted by determining the intersection between the differentially methylated genes (DMGs) and DEGs. A number of studies have shown that promoter methylation leads to the inactivation of tumor-suppressor genes, and thus is an important mechanism in the development of cancer.^33–35^ We noted that the DMGs were defined based on their promoter methylation status. A gene was considered to be differentially methylated if there was at least one DMC in its promoter region. The genes were classified into four groups based on the intersection between the DMGs and DEGs: hypermethylated–upregulated (hyper–up), hypermethylated–downregulated (hyper–down), hypomethylated–upregulated (hypo–up), and hypomethylated–downregulated (hypo–down) genes (Fig. 3a). Of the 2096 hypermethylated genes, 123 genes were upregulated and 601 genes were downregulated. Among the 1800 hypomethylated genes, 165 genes were upregulated and 203 genes were downregulated (Fig. 3b). For the downstream marker identification process, we focused on hyper–down genes, since the transcriptional silencing of tumor-suppressor genes by the aberrant hypermethylation of promoters is one of the most frequently observed alterations in cancers. GO analysis of the hyper–down genes revealed a significant enrichment of genes involved in chemical synaptic transmission, homophilic cell adhesion via plasma membrane adhesion molecules, nervous system development, the glutamate receptor signaling pathway, and potassium ion transmembrane transport (Fig. 3c). KEGG analysis demonstrated that five pathways were significantly enriched: neuroactive ligand–receptor interaction, circadian entrainment, calcium signaling, cell adhesion molecule, and retrograde endocannabinoid signaling (Fig. 3c, Table S2). Similar pathways were enriched in gene expression studies of various cancer types.^38,39^ Importantly, circadian rhythm disruption has been reported to be associated with several pathological conditions, including cancer progression.^40,41^ Both calcium signaling and cell adhesion molecule pathways are cancer-related pathways that may lead to cell proliferation or apoptosis.

Correlation analysis has been widely used to examine the relationship between methylation and gene expression.^42,43^ We examined the impact of DNA methylation on the local (the expression of a gene regulated by the promoter methylation of that gene) and distant regulation (the expression of a gene regulated by the promoter methylation of other genes) of gene expression by conducting Pearson correlation analysis. Of the 93,262 CpG-gene pairs analyzed in local regulation, 323 (10%) genes exhibited a significant positive correlation, 2973 (88%) genes showed a significant negative correlation, and 83 (2%) genes revealed both positive and negative correlations (Fig. 3d). We noted that a gene can be associated with CpGs both positively and negatively, since one gene can contain multiple CpGs in the promoter region. A high percentage of negative correlation was also observed in the correlation analysis of DMGs, DEGs, and differentially expressed and methylated genes (Fig. 3d). We then investigated the effect of DNA methylation on the distant regulation of gene expression. The Pearson correlation was calculated between 1092 CpGs in differentially expressed and methylated genes and 4949 DEGs. The promoter methylation of hypermethylated genes was more likely to be negatively correlated with gene expression, while the promoter methylation of hypomethylated genes was more likely to be positively correlated with gene expression (Fig. 3e).

### Identification and validation of cervical cancer-specific methylation markers

**Fig. 4:**
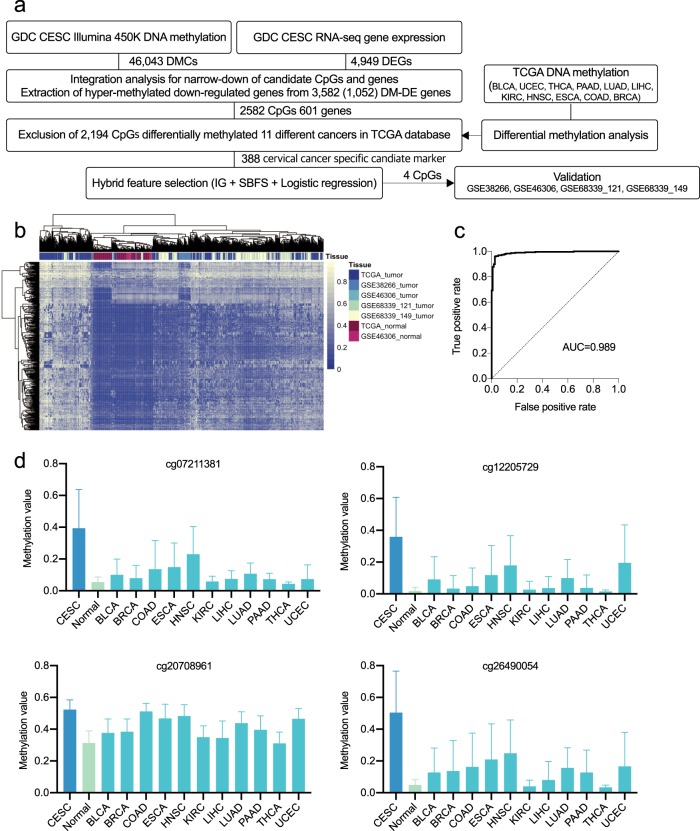
Identification of cervical cancer-specific biomarkers. **a** The workflow used to identify cervical cancer-specific methylation markers (GDC: Genomic Data Commons; CESC: cervical squamous cell carcinoma and endocervical adenocarcinoma). **b** Hierarchical clustering of the 388 candidate CpGs in samples from TCGA, GSE38266, GSE46306, and GSE68339. **c** Receiver-operating characteristic (ROC) curve and AUC values for TCGA data with tenfold cross-validation. **d** The distribution of the methylation levels for the four final selected markers in TCGA data of cervical tumors, normal tissues, and other cancers

To determine reliable cervical cancer-specific methylation signatures, we stringently screened the markers by using TCGA methylation data from other cancers and machine-learning techniques (Fig. 4a). Of the 2582 hypermethylated CpGs located in the promoter regions of the 601 downregulated genes, we excluded 2194 DMCs identified in 11 other types of cancers, including bladder cancer (BLCA), endometrioid cancer (UCEC), thyroid cancer (THCA), pancreatic cancer (PAAD), lung adenocarcinoma (LUAD), liver cancer (LIHC), clear-cell carcinoma (KIRC), head and neck cancer (HNSC), esophageal cancer (ESCA), colon cancer (COAD), and breast cancer (BRCA). We then performed hierarchical clustering of the remaining 388 CpGs from samples from TCGA, GSE38266, GSE46306, and GSE68339 (Fig. 4b). The normal samples were clustered together and well distinguished among the TCGA samples and other tumor samples in the validation sets. A hybrid feature selection approach based on information gain and sequential backward feature selection (SBFS) was adopted for further filtering of the candidate markers. Finally, we identified four cervical cancer-specific markers, cg07211381, cg12205729, cg20708961, and cg26490054. These CpGs were mapped to four different genes: RAB3C (cg07211381), GABRA2 (cg12205729), ZNF257 (cg20708961), and SLC5A8 (cg26490054). The distribution of the methylation levels of the four markers in cervical tumors, normal tissues, and other cancers clearly showed that the four identified CpGs were cervical cancer-specific markers and were not differentially methylated in other cancers (Fig. 4d).

We then built a logistic regression model by using the TCGA samples as a training set, and tenfold cross-validation was performed to achieve reliable predictive measurement. In the tenfold cross-validation, the data were randomly divided into ten different sets. Nine sets were used for training, and the remaining set was used for validation. The area under the curve (AUC) was 0.989, indicating that the four markers could achieve excellent performance in distinguishing TCGA cervical cancer and normal samples (Fig. 4c). To validate the markers in the independent sets, we tested the performance of the model on GSE38266, GSE46306, GSE68339_121, and GSE68339_149. Note that we chose the normal samples in GSE46306 as a control for GSE68339 and GSE38266, since these data only have tumor samples. We summarized the classification performance for the validation sets in terms of the true-positive (TP) rate, the false-positive (FP) rate, precision, the F-measure, and the AUC (Table 1a–d). The four markers could perfectly classify tumors from controls for GSE38266, GSE68339_121, and GSE68339_149. In contrast, the true-positive rate for GSE46306 was relatively lower than that for the other datasets due to the limited number (*n* = 6) of tumor samples, which made it difficult to determine accurate decision boundaries.

**Table 1:** (a) TP rate, FP rate, precision, F-measure, and AUC of logistic regression on GSE38266. (b) TP rate, FP rate, precision, F-measure, and AUC of logistic regression on GSE46306. (c) TP rate, FP rate, precision, F-measure, and AUC of logistic regression on GSE68339_121. (d) TP rate, FP rate, precision, F-measure, and AUC of logistic regression on GSE68339_149

	TP rate	FP rate	Precision	F-measure	AUC
(a)					
Tumor	1	0	1	1	1
Normal	1	0	1	1	1
Average	1	0	1	1	1
(b)					
Tumor	0.500	0	1	0.667	0.942
Normal	1	0.500	0.870	0.930	0.942
Average	0.885	0.385	0.900	0.869	0.942
(c)					
Tumor	1	0	1	1	1
Normal	1	0	1	1	1
Average	1	0	1	1	1
(d)					
Tumor	1	0	1	1	1
Normal	1	0	1	1	1
Average	1	0	1	1	1

The last row shows the weighted average of the performance

Next, we compared the four markers with known cervical cancer-specific markers in terms of classification accuracy and AUC (Table 2). We trained the logistic regression models by using 11 CpGs mapped to NOL4 and LHFPL4 according to Wang et al.^44^, 36 CpGs mapped to GHSR, SST, and ZIC1 according to Verlaat et al.^8^, 60 CpGs mapped to SOX1, PAX1, LMX1A, NKX6-1, WT1, and ONECUT1 according to Lai et al.^45^, and 37 CpGs mapped to DCC, EPB41L3, and SOX1 according to Clarke et al.^46^ (Table S3). The classification performances for the different marker sets were comparable, and the four markers identified in this study could achieve better performance for most validation datasets.

**Table 2:** (a) Comparison of AUCs of previously published cervical cancer-specific methylation markers for predicting tumor and normal tissues. (b) Comparison of classification accuracies of previously published cervical cancer-specific methylation markers for predicting tumor and normal tissues

	TCGA	GSE46306	GSE38266_42	GSE68339_121	GSE68339_149
(a)					
Wang et al.	0.977	0.758	0.969	0.978	0.980
Verlaat et al.	0.982	**1**	0.901	**1**	0.995
Lai et al.	0.978	**1**	0.572	0.987	**1**
Clarke et al.	0.967	0.295	0.337	0.454	0.472
This study	**0.989**	0.942	**1**	**1**	**1**
(b)					
Wang et al.	0.945	0.885	0.887	0.922	0.893
Verlaat et al.	0.948	0.962	0.839	0.986	0.982
Lai et al.	0.936	**1**	0.613	0.979	0.994
Clarke et al.	0.929	0.231	0.435	0.816	0.846
This study	**0.962**	0.885	**1**	**1**	**1**

### GABRA2, ZNF257, and SLC5A8 are weakly expressed in human CSCC

**Fig. 5:**
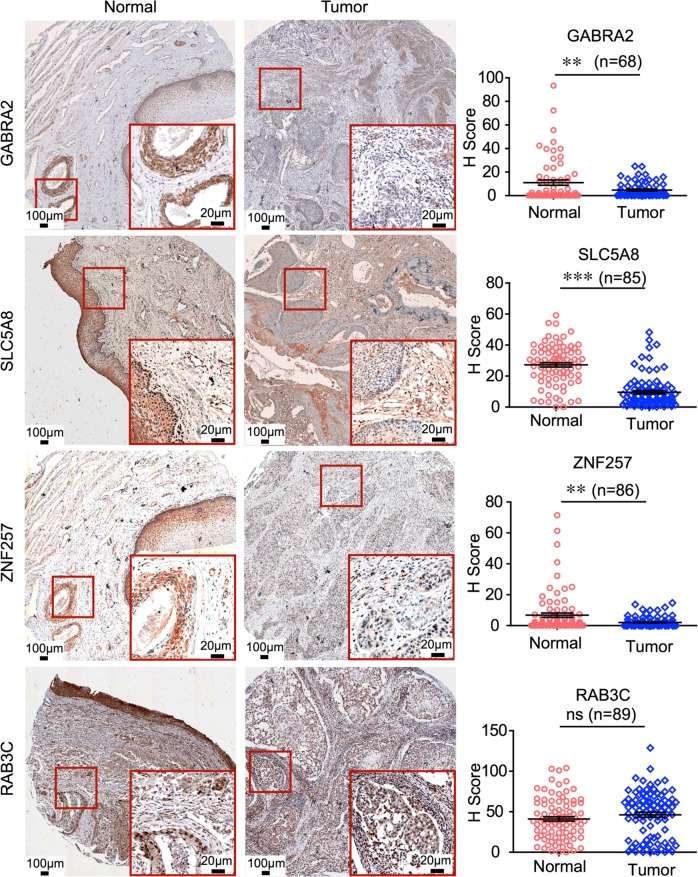
GABRA2, SLC5A8, and ZNF257 are weakly expressed in human cervical squamous cell carcinoma (CSCC). IHC staining of the indicated proteins in a human CSCC tissue array containing 93 intact cancer tissues and paired normal adjacent tissues. Representative images are shown in the left panels. Magnified images are shown in red boxes. The *H*-score-based quantification results are shown in the right panels. **p < 0.01, ***p < 0.001, Student’s *t* test

To further investigate the expression of the four newly identified hypermethylated cervical cancer-specific markers in human CSCC specimens, we assessed the protein levels of GABRA2, ZNF257, SLC5A8, and RAB3C by immunohistochemistry (IHC) using tissue arrays containing 93 human CSCC samples and paired cancer-adjacent normal tissues. We quantified IHC staining in CSCC specimens with a scoring scale (*H*-score) that combined the staining intensity and the percentage of positive cells. We found significantly lower levels of staining for GABRA2, ZNF257, and SLC5A8 in CSCC cells when compared with that in adjacent normal cells, whereas the level of RAB3C showed no difference between CSCC and normal cells (Fig. 5). These results strongly suggested that the hypermethylation of GABRA2, ZNF257, and SLC5A8 was correlated with the decreased expression of these genes in human CSCC cells, confirming that GABRA2, ZNF257, and SLC5A8 could be new diagnostic markers for human CSCC. We noted that the validation result for RAB3C was not consistent with that of the TCGA dataset, and further examination of methylation status and the mRNA expression levels of RAB3C is needed.

## Discussion

In this study, we analyzed DNA methylation and gene expression profiles in cervical cancer samples from TCGA. Unsupervised clustering analysis of DNA methylation profiles identified three methylation subtypes of cervical cancer, including CIMP-high, CIMP-intermediate, and CIMP-low. CIMP was initially identified in colorectal cancer and has been reported to be associated with poor prognosis in different types of cancer, such as gastric cancer and hepatocellular carcinoma.^47^ However, our survival analysis results (Fig. S1) indicated that CIMP was not associated with cervical cancer prognosis (*p*-value = 0.75). This is reasonable because most of the samples in the CIMP-high group were adenocarcinoma, and there is no evidence that adenocarcinoma results in poorer prognosis compared with other histological subtypes. The correlation analysis results revealed that promoter methylation was negatively correlated with the local regulation of gene expression. This observation is consistent with previous studies showing that promoter hypermethylation is associated with transcriptional silencing of genes. Interestingly, a negative correlation between promoter methylation and distant gene expression was observed in hypermethylated genes, while a positive correlation was found in hypomethylated genes. The detailed mechanism of the distant regulation of gene expression remains poorly understood, and further investigation is needed.

Feature selection is a data preprocessing technique that has been widely used in many bioinformatics applications.^48–50^ Here, we modeled marker discovery as a problem of selecting the best feature subset for the classification of cervical tumors and normal tissues. Choosing a reliable feature subset is not an easy task due to the high dimensionality of Illumina HumanMethylation450K data. It is impractical to use wrappers for marker discovery in DNA methylation studies because the computational cost is too expensive to test all possible combinations of 450-K CpGs. Applying filters to select markers is another challenge, since filters may lead to locally optimum sets but not the best discriminative subset, which may make it impossible to find diagnostic markers with high sensitivity and specificity. Therefore, we designed a hybrid feature selection schema based on information gain and SBFS to select reliable methylation markers. The results indicated that the proposed hybrid method performed extremely well for identifying methylation markers in cervical cancer.

We finally identified four methylation markers, cg07211381 (RAB3C), cg12205729 (GABRA2), cg20708961 (ZNF257), and cg26490054 (SLC5A8). By using these four markers, we achieved 96.2% sensitivity and 95.2% specificity in the tenfold cross-validation of TCGA data. Moreover, we obtained 94.2, 100, 100, and 100% AUCs for four independent validation sets from the GEO database. One of the marker genes, SLC5A8, a tumor-suppressor gene, was previously found to be hypermethylated in colon neoplasia,^51^ and the downregulation of SLC5A8 caused by aberrant DNA methylation was observed in breast cancer cells.^52^ The remaining three genes, RAB3C, GABRA2, and ZNF257, have not been reported to be associated with cancers, and further investigation of their biological functions is needed.

In summary, DNA methylation is a major epigenetic mechanism that plays a crucial role in carcinogenesis. We identified four cervical cancer-specific methylation markers, including cg07211381 (RAB3C), cg12205729 (GABRA2), cg20708961 (ZNF257), and cg26490054 (SLC5A8), and the significantly decreased expression of GABRA2, ZNF257, and SLC5A8 in CSCC was further confirmed in human CSCC tissues by IHC. The experimental results indicated that these markers have extremely high sensitivity and specificity in distinguishing cervical tumors and normal tissues. Further biological validation and clinical trials are needed to evaluate the clinical significance of these methylation markers. Furthermore, we believe that our study can shed light on the cellular and biological mechanisms of cervical cancer development and oncogenesis and help to improve early detection and early intervention for cancers in the clinic, especially for slow-growing but easily metastasized cancers such as cervical cancer.

## Materials and methods

### Data preparation

Illumina HumanMethylation450K array data from 307 cervical tumors and 3 associated normal tissues were obtained from TCGA, and the methylation levels were quantified as beta values (*β*), which are the ratio of the intensities of methylated and unmethylated alleles. We extracted HumanMethylation450K array data from 110 normal samples from 11 other cancer projects by randomly selecting ten samples from each project, as described in ref. ^1^ The 11 cancer types included BLCA, UCEC, THCA, PAAD, LUAD, LIHC, KIRC, HNSC, ESCA, COAD, and BRCA. The methylation profiles of 113 normal samples were finally used as controls for differential methylation analysis. The probes were annotated by using the Bioconductor package with the human genome assembly GRCh37 (hg19). In addition, four Illumina 450K methylation datasets were obtained from the GEO database. GSE38266^53^ includes the methylation data from 21 HPV-positive and 21 HPV-negative tumors, GSE46306^37^ contains data from 20 normal cervical samples (HPV-negative) and 6 cervical cancer tissues (HPV-positive), and GSE68339^54^ contains methylation profiles from a discovery cohort of 149 cervical cancer patients (GSE68339_149) and a validation cohort of 121 cancer patients (GSE68339_121). RNA-seq expression profiles from cervical cancers were also obtained from TCGA. Raw read counts from 304 tumors and 69 normal tissues matching the methylation data were included in the study.

### Consensus clustering analysis

Unsupervised consensus clustering of the 591 most variable probes in CpG island (CGI) promoter regions was performed according to the K-means algorithm and Euclidean distance. To determine the most variable CpGs, we selected the CpGs in 307 cervical cancer samples with a standard deviation for the *β* value that was larger than 0.2 and removed the CpG if the average methylation level in 113 normal tissues was larger than 0.05, as described in previous studies.^55,56^ The Consensus Cluster Plus R package^57^ was used for the clustering analysis. Fisher’s exact test was used to test the significance of the clinical features across the clusters, and one-way ANOVA was performed to compare the methylation levels of CpGs among different clusters.

### Differential methylation analysis

Differential methylation analysis was performed between 113 normal and 307 tumor samples. Probes containing SNPs, probes in chromosome X, and probes with more than 10% missing values were excluded from the analysis (Additional File 1). The missing values of the selected CpGs were imputed by using the imputeTS R package. The Wilcoxon rank-sum test was used to determine the differentially methylated CpGs (DMCs), and the p-values were adjusted by using the FDR method. DMCs were reported if the mean methylation difference was >0.2 with an FDR of 5%.

### Differential expression analysis and DAVID analysis

Among the 113 normal and 307 tumor samples used for the methylation analysis, 69 normal and 304 tumor samples with RNA-seq expression profiles were included in the differential expression analysis. The trimmed mean of M values (TMM) method was used to normalize the raw RNA-seq read counts, and the negative binomial generalized log-linear model was used to fit the normalized counts. Differentially expressed genes (DEGs) were reported if the log-fold change was >1.5 and the adjusted p-value was smaller than 0.05. Functional annotation clustering analysis was performed by using the Database for Annotation, Visualization, and Integrated Discovery (DAVID).^58^ The top five gene ontology (GO) biological processes and Kyoto Encyclopedia of Genes and Genomes (KEGG) pathways were reported with their *p*-values and Benjamini–Hochberg^59^ values.

### Correlation analysis between CpGs and genes

To examine the impact of DNA methylation on the local regulation of gene expression, the Pearson correlation (*r*) was calculated between the *β* values of CpGs located in promoter regions and the normalized expression values of the corresponding genes. Note that a gene can be linked via multiple CpGs in its promoter, and thus, the correlation was calculated for each CpG-gene pair. $|r|$ >0.3 and an adjusted *p*-value <0.05 were set as the cutoffs for a significant correlation.^60^ To investigate the distant regulation of gene expression, *r* was calculated between the *β* values of CpGs of differentially methylated and expressed genes and the normalized expression values of differentially expressed genes.

### Hybrid feature selection

Feature selection is a preprocessing technique in machine learning used to reduce the dimensionality of data. The goal of feature selection is to identify the most informative subset of features that leads to better learning performance. Feature selection approaches can be broadly divided into three categories: filter, wrapper, and hybrid.^61^ In filter methods, the features are ranked by a score calculated from the statistical measures, and a cutoff value is provided to determine whether a feature should be selected. Wrapper methods generate different combinations of features and evaluate them by using a machine-learning algorithm. Hybrid methods first eliminate some features by using filters and then apply wrappers to determine the final subset. The hybrid approach not only takes advantage of the computational efficiency of filters but can also achieve comparable accuracy to that of wrappers.

To identify reliable methylation markers of cervical cancer, we viewed marker discovery as a problem of identifying a feature (CpG) subset that can most precisely discriminate cervical tumors and normal tissues. We designed a hybrid feature selection schema based on information gain and sequential backward feature selection (SBFS). First, the information gain of all candidate CpGs was calculated as follows:

$${\mathrm{IG}} = H\left( X \right) + H(X|Y)$$

where *H*(*X*) denotes the entropy of the CpG X and *H*(*X/Y*) indicates the entropy of the CpG X after observing class *Y*. All the CpGs were then sorted in descending order according to their information gain, and a threshold was established to remove irrelevant CpGs. If the information gain of a CpG was >0.3, the CpG was selected for further processing; if not, the CpG was eliminated. Next, different combinations of CpGs were generated by using SBFS, and a logistic regression model was used to evaluate the combinations. The subset with the highest classification accuracy was selected as the final subset.

### Immunohistochemistry

Consecutive sections from three human cervical squamous cell carcinoma tissue arrays containing 93 intact cervical carcinoma tissues and paired normal adjacent cervical tissues were purchased from Shanghai Outdo Biotech Co., Ltd. (OD-CT-RpUtr03-004, OD-CT-RpUtr03-005, and OD-CT-RpUtr03-006). The sections were stained with anti-GABRA2 antibody (Thermo-Fisher, #PA5-26305) at a 1:100 dilution, anti-ZNF257 antibody (Thermo-Fisher, #PA5-36012) at a 1:100 dilution, anti-SLC5A8 antibody (Proteintech, #21433-1-AP) at a 1:100 dilution, and anti-RAB3C antibody (Proteintech, #10788-1-AP) at a 1:200 dilution. After washing, the sections were incubated with biotin-conjugated secondary antibodies and subsequently with streptavidin–HRP. The sections were finally visualized by incubation with 3,3′-diaminobenzidine (DAB) substrate. Images were obtained with the Mantra System (PerkinElmer, Massachusetts, USA) with identical exposure times. The *H*-score was used for quantifying the protein levels of GABRA2, ZNF257, SLC5A8, and RAB3C in normal and tumor tissues, and this score was calculated by multiplying the staining area (scored as the percentage of differentially stained area) and the staining intensity (weak, moderate, and strong were scored as 1, 2, and 3 based on color density). Student’s *t* test was performed for the statistical analysis.

## Supplementary information

Supplemental Material

Additional File 1

## Supplementary information

**Supplementary information** The online version of this article (10.1038/s41392-019-0081-6) contains supplementary material, which is available to authorized users.
