# Supplementary material for: Integrative analysis of DNA methylation and gene expression identified cervical cancer-specific diagnostic biomarkers
Source: Signal Transduct Target Ther. 2019 Dec 13;4:55. doi: 10.1038/s41392-019-0081-6 (PMC6908647; doi:10.1038/s41392-019-0081-6)
Supplement: Supplementary file 1 — Supplemental Material [file 41392_2019_81_MOESM1_ESM.docx]

Supplementary Materials for

Integrative analysis of DNA methylation and gene expression identifies cervical cancer-specific diagnostic biomarkers

Wanxue Xu^1^, Mengyao Xu^1^, Longlong Wang^1,3^, Wei Zhou^1^, Rong Xiang^1^, Yi Shi^1,3,*^, Yunshan Zhang^2,*^, Yongjun Piao^1,3,*^

^1^School of Medicine, Nankai University, Tianjin, China

^2^Reproductive Medical Center, Nankai University affiliated Hospital of Obstetrics and Gynecology, Tianjin, China

^3^Tianjin Key Laboratory of Human Development and Reproductive Regulation, Nankai University affiliated Hospital of Obstetrics and Gynecology, Tianjin, China

^*^Correspondence to: Yongjun Piao (ypiao@nankai.edu.cn), Yunshan Zhang (tjzys@hotmail.com) and Yi Shi (yishi@nankai.edu.cn)

Phone: (86)-22-23509482

Fax: (86)-22-23502554

Conflicts of interests: The authors declare no competing interests.

**This PDF file includes:**

Figures. S1

Tables S1 to S3

**Other Supplementary Materials for this manuscript include the following:**

Additional File 1. List of excluded probes contain SNPs.


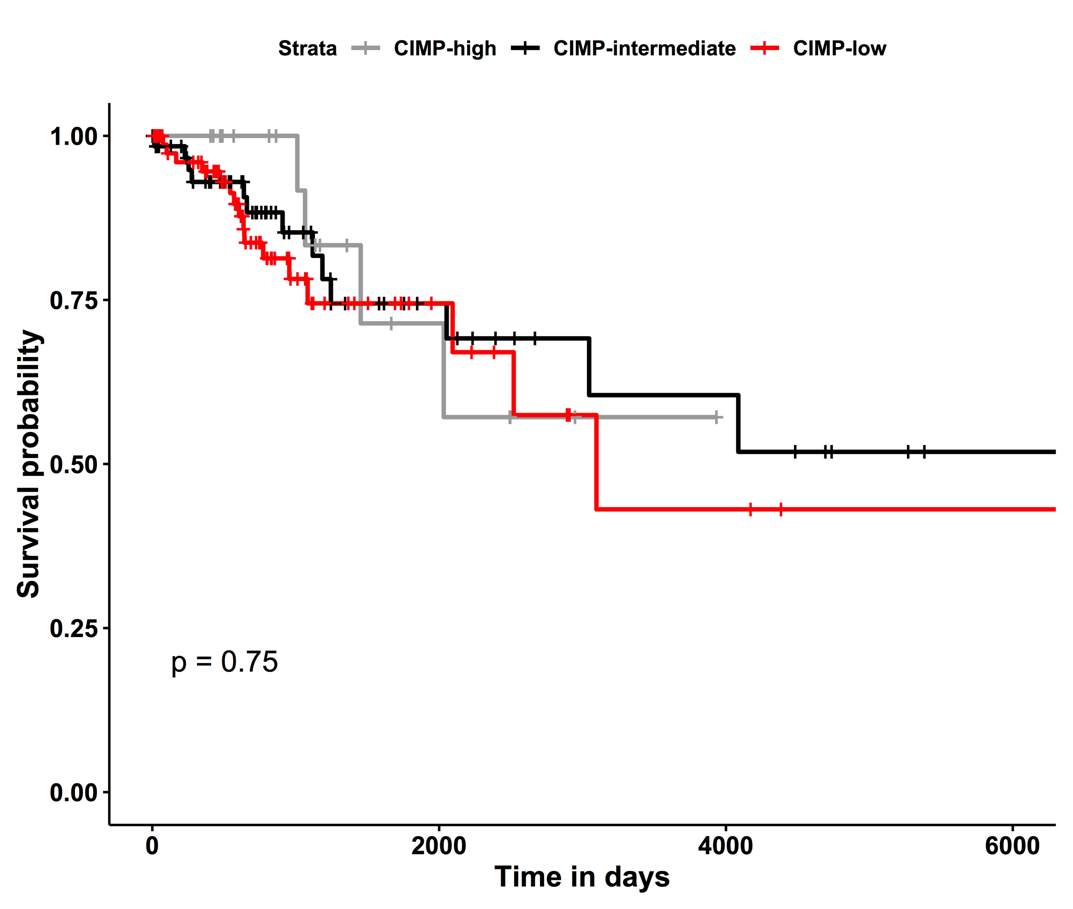


Figure. S1.

Kaplan-Meier survival curves of each cluster. The identified clusters were CIMP-high (n=20, CpG island methylator phenotype), CIMP-intermediate (n=69) and CIMP-low (n=89).

Table S1.

List of TCGA sample id for 178 well-annotated samples.

| TCGA-BI-A0VR-01A | TCGA-C5-A3HE-01A | TCGA-EA-A44S-01A | TCGA-EK-A3GN-01A | TCGA-JW-A5VH-01A |
| --- | --- | --- | --- | --- |
| TCGA-BI-A0VS-01A | TCGA-C5-A3HF-01A | TCGA-EA-A4BA-01A | TCGA-EX-A1H5-01A | TCGA-JW-A5VI-01A |
| TCGA-BI-A20A-01A | TCGA-C5-A3HL-01A | TCGA-EA-A50E-01A | TCGA-EX-A1H6-01B | TCGA-JW-A5VJ-01A |
| TCGA-C5-A0TN-01A | TCGA-C5-A7CG-01A | TCGA-EA-A556-01A | TCGA-EX-A3L1-01A | TCGA-JW-A5VK-01A |
| TCGA-C5-A1BE-01B | TCGA-C5-A7CH-01A | TCGA-EA-A5FO-01A | TCGA-EX-A69L-01A | TCGA-JW-A5VL-01A |
| TCGA-C5-A1BF-01B | TCGA-C5-A7CJ-01A | TCGA-EA-A5O9-01A | TCGA-EX-A69M-01A | TCGA-JW-A69B-01A |
| TCGA-C5-A1BI-01B | TCGA-C5-A7CK-01A | TCGA-EA-A5ZD-01A | TCGA-FU-A23K-01A | TCGA-JX-A3PZ-01A |
| TCGA-C5-A1BJ-01A | TCGA-C5-A7CL-01A | TCGA-EA-A5ZE-01A | TCGA-FU-A23L-01A | TCGA-JX-A3Q0-01A |
| TCGA-C5-A1BK-01B | TCGA-C5-A7CM-01A | TCGA-EA-A5ZF-01A | TCGA-FU-A2QG-01A | TCGA-JX-A3Q8-01A |
| TCGA-C5-A1BL-01A | TCGA-C5-A7UE-01A | TCGA-EA-A6QX-01A | TCGA-FU-A3EO-01A | TCGA-JX-A5QV-01A |
| TCGA-C5-A1BM-01A | TCGA-DG-A2KH-01A | TCGA-EA-A78R-01A | TCGA-FU-A3HY-01A | TCGA-LP-A4AU-01A |
| TCGA-C5-A1BN-01B | TCGA-DG-A2KJ-01A | TCGA-EK-A2GZ-01A | TCGA-FU-A3NI-01A | TCGA-LP-A4AV-01A |
| TCGA-C5-A1BQ-01C | TCGA-DG-A2KK-01A | TCGA-EK-A2H0-01A | TCGA-FU-A3TQ-01A | TCGA-LP-A4AW-01A |
| TCGA-C5-A1M5-01A | TCGA-DG-A2KL-01A | TCGA-EK-A2H1-01A | TCGA-FU-A3TX-01A | TCGA-LP-A4AX-01A |
| TCGA-C5-A1M6-01A | TCGA-DG-A2KM-01A | TCGA-EK-A2IP-01A | TCGA-FU-A3WB-01A | TCGA-LP-A5U2-01A |
| TCGA-C5-A1M7-01A | TCGA-DR-A0ZL-01A | TCGA-EK-A2PG-01A | TCGA-FU-A3YQ-01A | TCGA-LP-A5U3-01A |
| TCGA-C5-A1M8-01A | TCGA-DR-A0ZM-01A | TCGA-EK-A2PI-01A | TCGA-FU-A40J-01A | TCGA-LP-A7HU-01A |
| TCGA-C5-A1M9-01A | TCGA-DS-A0VK-01A | TCGA-EK-A2PK-01A | TCGA-FU-A57G-01A | TCGA-MU-A51Y-01A |
| TCGA-C5-A1ME-01A | TCGA-DS-A0VL-01A | TCGA-EK-A2PL-01A | TCGA-FU-A5XV-01A | TCGA-MU-A5YI-01A |
| TCGA-C5-A1MF-01A | TCGA-DS-A0VM-01A | TCGA-EK-A2PM-01A | TCGA-FU-A770-01A | TCGA-MY-A5BD-01A |
| TCGA-C5-A1MH-01A | TCGA-DS-A0VN-01A | TCGA-EK-A2R7-01A | TCGA-HG-A2PA-01A | TCGA-MY-A5BE-01A |
| TCGA-C5-A1MI-01A | TCGA-DS-A3LQ-01A | TCGA-EK-A2R8-01A | TCGA-HM-A3JJ-01A | TCGA-MY-A5BF-01A |
| TCGA-C5-A1MJ-01A | TCGA-DS-A5RQ-01A | TCGA-EK-A2R9-01A | TCGA-HM-A3JK-01A | TCGA-Q1-A5R1-01A |
| TCGA-C5-A1MK-01A | TCGA-EA-A1QS-01A | TCGA-EK-A2RA-01A | TCGA-HM-A4S6-01A | TCGA-Q1-A5R2-01A |
| TCGA-C5-A1ML-01A | TCGA-EA-A1QT-01A | TCGA-EK-A2RB-01A | TCGA-HM-A6W2-01A | TCGA-Q1-A5R3-01A |
| TCGA-C5-A1MN-01A | TCGA-EA-A3HQ-01A | TCGA-EK-A2RC-01A | TCGA-IR-A3L7-01A | TCGA-Q1-A6DT-01A |
| TCGA-C5-A1MP-01A | TCGA-EA-A3HR-01A | TCGA-EK-A2RE-01A | TCGA-IR-A3LA-01A | TCGA-Q1-A6DV-01A |
| TCGA-C5-A2LS-01A | TCGA-EA-A3HT-01A | TCGA-EK-A2RJ-01A | TCGA-IR-A3LB-01A | TCGA-Q1-A6DW-01A |
| TCGA-C5-A2LT-01A | TCGA-EA-A3HU-01A | TCGA-EK-A2RK-01A | TCGA-IR-A3LC-01A | TCGA-Q1-A73O-01A |
| TCGA-C5-A2LV-01A | TCGA-EA-A3QD-01A | TCGA-EK-A2RL-01A | TCGA-IR-A3LF-01A | TCGA-Q1-A73P-01A |
| TCGA-C5-A2LX-01A | TCGA-EA-A3QE-01A | TCGA-EK-A2RM-01A | TCGA-IR-A3LH-01A | TCGA-Q1-A73Q-01A |
| TCGA-C5-A2LY-01A | TCGA-EA-A3Y4-01A | TCGA-EK-A2RN-01A | TCGA-IR-A3LI-01A | TCGA-Q1-A73R-01A |
| TCGA-C5-A2LZ-01A | TCGA-EA-A410-01A | TCGA-EK-A2RO-01A | TCGA-IR-A3LK-01A | TCGA-Q1-A73S-01A |
| TCGA-C5-A2M1-01A | TCGA-EA-A411-01A | TCGA-EK-A3GJ-01A | TCGA-IR-A3LL-01A | TCGA-R2-A69V-01A |
| TCGA-C5-A2M2-01A | TCGA-EA-A439-01A | TCGA-EK-A3GK-01A | TCGA-JW-A5VG-01A | TCGA-RA-A741-01A |
| TCGA-C5-A3HD-01B | TCGA-EA-A43B-01A | TCGA-EK-A3GM-01A |  |  |

Table S2.

DAVID functional analysis results for hyper-up, hypo-up, and hypo-down genes.

| **Hyper-upregulated genes - GO: biological process** | **P-value** | **Benjamini** |
| --- | --- | --- |
| positive regulation of transcription from RNA polymerase II promoter | 1.20E-07 | 1.10E-04 |
| negative regulation of transcription from RNA polymerase II promoter | 1.40E-06 | 6.40E-04 |
| transcription from RNA polymerase II promoter | 1.80E-06 | 5.30E-04 |
| negative regulation of neuron apoptotic process | 3.00E-06 | 6.70E-04 |
| proximal/distal pattern formation | 1.90E-05 | 3.30E-03 |
| **Hypo-upregulated genes - GO: biological process** | **P-value** | **Benjamini** |
| epidermis development | 2.40E-13 | 2.10E-10 |
| hemidesmosome assembly | 2.20E-06 | 9.60E-04 |
| keratinization | 2.80E-06 | 8.20E-04 |
| keratinocyte differentiation | 4.10E-05 | 9.00E-03 |
| establishment of skin barrier | 4.20E-04 | 7.20E-02 |
| **Hypo-downregulated genes - GO: biological process** | **P-value** | **Benjamini** |
| muscle contraction | 1.50E-05 | 1.40E-02 |
| cellular response to drug | 7.20E-04 | 2.80E-01 |
| regulation of postsynaptic membrane potential | 1.40E-03 | 3.60E-01 |
| cellular response to tumor necrosis factor | 5.60E-03 | 7.30E-01 |
| cellular response to interleukin-1 | 6.20E-03 | 6.80E-01 |
| **Hyper-upregulated genes - KEGG: pathway** | **P-value** | **Benjamini** |
| Signaling pathways regulating pluripotency of stem cells | 1.70E-03 | 1.10E-01 |
| GABAergic synapse | 1.90E-03 | 6.20E-02 |
| Morphine addiction | 1.90E-02 | 3.50E-01 |
| Retrograde endocannabinoid signaling | 2.50E-02 | 3.50E-01 |
| Nicotine addiction | 2.60E-02 | 3.00E-01 |
| **Hypo-upregulated genes - KEGG: pathway** | **P-value** | **Benjamini** |
| Amoebiasis | 2.00E-04 | 2.90E-02 |
| Rheumatoid arthritis | 5.50E-03 | 3.30E-01 |
| Alcoholism | 1.40E-02 | 5.00E-01 |
| p53 signaling pathway | 1.70E-02 | 4.60E-01 |
| Cell cycle | 1.80E-02 | 4.10E-01 |
| **Hypo-downregulated genes - KEGG: pathway** | **P-value** | **Benjamini** |
| Neuroactive ligand-receptor interaction | 1.40E-02 | 8.60E-01 |
| Salivary secretion | 1.70E-02 | 7.10E-01 |
| Protein digestion and absorption | 1.80E-02 | 5.80E-01 |
| Cell adhesion molecules (CAMs) | 8.00E-02 | 9.50E-01 |
| - | - | - |

Table S3.

Previously published methylation markers in cervical cancer.

| Wang et al. 2008 | Verlaat et al. 2017 | Lai et al. 2008 | Clarke et al. 2017 |
| --- | --- | --- | --- |
| cg00884606 | cg22070855 | cg08460041 | cg21381065 |
| cg26490054 | cg24599434 | cg17620199 | cg12967001 |
| cg24248713 | cg14741939 | cg03140968 | cg06550462 |
| cg22287067 | cg18424634 | cg08448701 | cg23564700 |
| cg03045425 | cg02647941 | cg19054524 | cg20829347 |
| cg08195448 | cg15987088 | cg01783070 | cg25122395 |
| cg18114671 | cg14768785 | cg20829347 | cg16747564 |
| cg06609489 | cg21463380 | cg16747564 | cg00027400 |
| cg12911428 | cg11812218 | cg25463470 | cg23847381 |
| cg16158843 | cg07120369 | cg06675478 | cg07935012 |
| cg00242951 | cg08855449 | cg24604013 | cg25463470 |
|  | cg23500122 | cg00663972 | cg16622495 |
|  | cg23468878 | cg27301032 | cg06675478 |
|  | cg22459146 | cg02547394 | cg13034362 |
|  | cg17152757 | cg19407095 | cg24604013 |
|  | cg15002294 | cg06657050 | cg00663972 |
|  | cg07848409 | cg12401926 | cg27301032 |
|  | cg13206017 | cg11199713 | cg02547394 |
|  | cg00475509 | cg02061705 | cg19407095 |
|  | cg12965599 | cg26463200 | cg02835371 |
|  | cg23449696 | cg13450005 | cg11199713 |
|  | cg17280346 | cg22058250 | cg14222939 |
|  | cg26400885 | cg26158897 | cg07897248 |
|  | cg16636671 | cg05456921 | cg16964748 |
|  | cg09241332 | cg20056542 | cg14743291 |
|  | cg26014538 | cg24495684 | cg19042459 |
|  | cg04851268 | cg13663793 | cg01757312 |
|  | cg21526749 | cg19126300 | cg18874136 |
|  | cg14703224 | cg13877670 | cg15528411 |
|  | cg06737494 | cg16426339 | cg06784848 |
|  | cg05700079 | cg19570244 | cg22335490 |
|  | cg10107050 | cg11114873 | cg18801691 |
|  | cg16181396 | cg04351049 | cg20194811 |
|  | cg07291439 | cg01757312 | cg21263710 |
|  | cg14456683 | cg05899118 | cg19613722 |
|  | cg01227537 | cg10103187 | cg25204852 |
|  |  | cg07686479 | cg01673082 |
|  |  | cg01952234 |  |
|  |  | cg09248345 |  |
|  |  | cg02847948 |  |
|  |  | cg22837767 |  |
|  |  | cg08606911 |  |
|  |  | cg02361878 |  |
|  |  | cg10539507 |  |
|  |  | cg20194811 |  |
|  |  | cg08593261 |  |
|  |  | cg07193766 |  |
|  |  | cg15897645 |  |
|  |  | cg09069138 |  |
|  |  | cg02054776 |  |
|  |  | cg26359204 |  |
|  |  | cg27440715 |  |
|  |  | cg01196531 |  |
|  |  | cg08739648 |  |
|  |  | cg04811512 |  |
|  |  | cg18813158 |  |
|  |  | cg15446043 |  |
|  |  | cg23971170 |  |
|  |  | cg06441568 |  |
|  |  | cg18589016 |  |
